# Socioeconomic Impact of COVID-19 on Spinal Instrumentation Companies in the Era of Decreased Elective Surgery

**DOI:** 10.7759/cureus.9776

**Published:** 2020-08-16

**Authors:** Brian Fiani, Ryne Jenkins, Imran Siddiqi, Asif Khan, Ashley Taylor

**Affiliations:** 1 Neurosurgery, Desert Regional Medical Center, Palm Springs, USA; 2 College of Osteopathic Medicine, Western University of Health Sciences, Pomona, USA; 3 Podiatry, Beaumont Hospital, Farmington Hills, USA; 4 Psychological, Health, and Learning Sciences, University of Houston, Houston, USA

**Keywords:** socioeconomics, spine surgery, financial, instrumentation companies, covid-19

## Abstract

Since the onset of the novel coronavirus (COVID-19) global health crisis, there has been an unprecedented change to the field of spinal surgery. Comprehensive protocols and algorithms have been implemented globally to maximize available bed space, conserve personal protective equipment, and to minimize exposure. This has resulted in a sharp decline in elective spinal surgery and placed an undue burden on the spinal industry. As the landscape of elective surgery changes, this paper looks to analyze the effects the COVID-19 pandemic has and will have on spinal instrumentation companies, surgeons, and the spinal industry. Changes in government policies, patient care, financial markets, and distribution have all presented an unprecedented strain on spinal instrumentation companies. A narrative literature review was performed using published literature from PubMed. Due to the socioeconomic and financial nature of this review, data collection from financial references was also obtained and cited. With significant financial losses reported throughout the spinal industry and medical field, this paper discusses managing the COVID-19 pandemic and the future prospectus moving forward. As the pandemic continues to unfold, it remains difficult to predict the exact timing for broad resumption of elective medical procedures, and the extent to which the pandemic will affect the industry. Preparation aimed at facilitating efficient resource allocation and communication among surgeons, surgical instrumentation representatives and hospital leadership is essential as we transition forward and reestablish normalcy under the new constraints of COVID-19.

## Introduction and background

Novel coronavirus 2019 (COVID-19) was declared an emergency of public concern by the World Health Organization on January 30, 2020, and was declared a pandemic on March 11, 2020 [1]. COVID-19’s pathogen, severe acute respiratory syndrome coronavirus 2 (SARS-CoV-2), which was first reported in Wuhan, China, belongs to the Orthocoronavirinae family [2]. To limit the spread of disease, several measures were set in place worldwide, including, but not limited to, social distancing, closure of public entities, and stay-at-home orders. In the context of the medicine field, many hospitals closed outpatient facilities and canceled/postponed elective procedures in order to limit the spread of the disease. With elective patients accounting for nearly 30% of hospital revenue, hospitals have experienced a drastic decrease in revenue, leading to lay-offs, furloughs, and protocols to cut costs [3].

During the 12 week peak of COVID-19, it was projected that 28,404,603 elective cases were canceled globally [4]. Models have also projected that countries would have to increase their surgical volume by 20% for 45 weeks to clear the backlog of elective cases [4]. Along with hospitals, spinal instrumentation companies have also been socioeconomically affected. Large spinal instrumentation manufactures have reported losses as high as 7.5 percent in the first quarter of 2020, largely attributed to the drop in elective cases due to the COVID-19 pandemic [5]. As the landscape of elective surgery changes, this paper looks to analyze the effects this pandemic has and will have on spinal instrumentation companies, both socially and financially.

## Review

Protocol for performing spine surgery during the COVID-19 era

Since the onset of the COVID-19 global health crisis, there has been a sharp decline in both orthopedic and spine surgeries. As hospitals experienced rapid overcrowding by COVID-19 patients, many spinal surgeons were called upon to care for these individuals, and a recent study found that one in four surgeons reported working outside of their typical scope of practice [6]. Coinciding with this significant shift in clinical role were the cancellations of elective surgery cases as well as the postponement of interventional spinal procedures and surgeries. Reasons for such cancellations are two-fold: to decrease patient and provider exposure to COVID-19, and conserving personal protective equipment and other valuable resources. In turn, surgeons were encouraged to focus their efforts on the treatment of patients requiring immediate care.

On April 22, 2020, the North American Spine Society (NASS) released a guidance document inclusive of clinical considerations and recommendations for surgeries and procedures within the following categories: emergent, urgent, and elective [7]. Patients requiring emergent care include those who are at risk of neurologic injury due to spinal instability, as well as those who require surgical decompression (i.e., to care for an epidural abscess). Likewise, emergent surgery is imperative for individuals who are experiencing neurologic compression which may result in progressive or severe neurologic deficits if left untreated and, according to the NASS guidance document, it is not advisable to postpone emergent procedures and/or treatments [7]. Patients requiring urgent care include those who are experiencing severe pain from nerve compression, as well as those who are at risk of losing important neurological functions. Urgent surgery is often performed to prevent neurological impairment and, in the wake of the COVID-19 global health crisis, is permissible if local conditions, policies, and regulations allow. In addition, the patient’s condition (i.e., overall health status), the availability of staff and personal protective equipment, and the current and projected cases of COVID-19 in the region and the facility where the procedures would be performed should be taken into consideration when deciding to perform emergent or urgent surgery [7].

Since the onset of COVID-19, elective surgeries such as spine fusion, total joint replacements, and chronic joint conditions have been delayed and postponed to maximize available bed space, conserve personal protective equipment, and to limit unnecessary exposure [8]. That said, in April 2020, new guidelines have been released making it permissible for several states to resume elective procedures [9-11]. However, for many patients whose pain can be managed reasonably without procedural intervention, elective procedures continue to be delayed while surgeons catch up.

As new information related to COVID-19 continues to emerge, the safety of patients, hospital staff, and providers remains the highest priority. As a response, there has been a plethora of communications and treatment recommendations published by international, federal, state, and local governing bodies. Conversely, comprehensive protocols and algorithms aimed at facilitating efficient resource allocation and communication across disciplines in crisis settings were lacking. To address these issues and issues related to the complexities of scheduling and preparing for surgeries, ensuring proper staffing, and limiting undue and unnecessary exposure to COVID-19, the Department of Neurological Surgery at the University of California San Francisco created a detailed checklist for booking neurosurgical cases, as well as a detailed neurosurgical treatment algorithm [12]. Both the checklist and treatment algorithm are flexible - something that is important to consider as the situation centering on COVID-19 remains rapidly evolving and ever-changing.

Impact on company financials

Due to the COVID-19 outbreak, there have been severe disruptions to the global economy, including to major companies in the spinal instrumentation and implant market. Governmental mitigation strategies, including quarantines, shelter-in-place orders, restrictions on travel, and social distancing have led to an economic slowdown that may continue for a prolonged duration. Companies have attempted to put in place procedures and measures to ensure the health safety of employees, and taken action to reduce operating expenses [13]. A large number of global suppliers, manufacturing facilities, and distributors have been adversely affected in several regions from the United States to China. Reduced availability of air transport, port closures, increased border controls, transportation costs, and security threats to the supply chain have contributed to an already increased volatile global market [14]. As a result of restrictive measures in response to the pandemic, operations for companies in the spine instrumentation market have been adversely affected. Several of these companies have products that are sensitive to the reductions in elective medical procedures, which were suspended in diverse markets during the first quarter of 2020, negatively impacting business, cash flows, and financial conditions.

It is difficult to predict the exact timing for broad resumption of elective medical procedures, and the extent to which people will need to de-prioritize, delay, or cancel elective procedures as a result of the COVID-19 pandemic. Other challenges that the spinal instrumentation companies have faced is delay in or shortage of supply of components and materials, as well as delivery of products. This has created a difficulty in the ability to satisfy consumer demands for products, or deliver them in a timely fashion, which could ultimately harm company reputation, profitability, and sales. Some companies rely on indirect distribution channels to market, distribute, and sell their products, and oftentimes these entities are the main point of contact for healthcare professionals and organizations. As a result of travel restrictions, and governmental shutdowns there is increased risk for companies if these indirect channels become insolvent, as their ability to market, distribute, and sell products will be impacted negatively. Additionally, companies have had to adjust operations as the market demand for certain products has shifted and continues to shift as mandates by government authorities in the response to the COVID-19 pandemic change.

**Figure 1:**
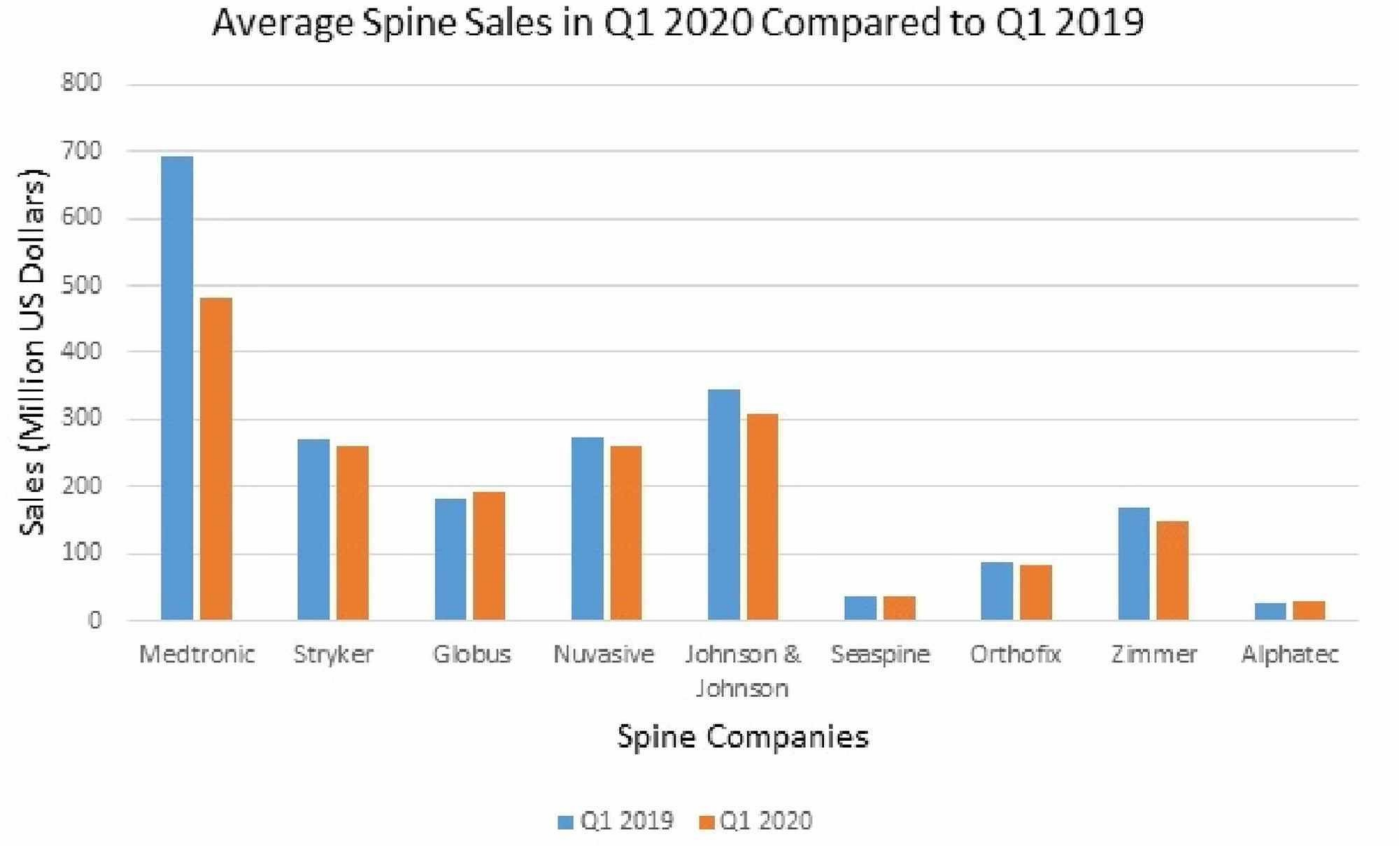
Impact of COVID-19 on the average spine instrumentation sales in Q1 2020 compared to sales in Q1 2019 of major spinal instrumentation companies

Overall, the average loss of nine main spine companies in the first quarter (Q1) of 2020 was $282 million, a 13.6% decline compared to Q1 of 2019 (Table 1, Figure 1). Zimmer Biomet Holdings, Inc. (Zimmer) reported Q1 net sales of $1.784 billion, a 9.7% decrease from 2019, and 8.9% decline on a constant currency basis [15]. Net loss in Q1 was $509 million, and net earnings were $354 million on an adjusted basis. The dramatic decrease in net sales was attributed to the majority of net sales being from products utilized in elective surgeries, a common theme reflected in the majority of quarterly reports for Q1 2020. Zimmer took steps in order to maintain an adequate financial profile to have access to capital in order to fund the business. Late in 2019, they put in place a restructuring plan to reduce costs, have temporarily reduced compensation for many employees, and have implemented temporary suspension or limited production in certain manufacturing facilities. Additionally, they have begun the utilization of government wage assistance programs in certain markets, including tax relief provisions in the United States (CARES Act). They expect to be impacted most significantly in Q2, with likely improvements in Q3 and Q4, although this is ultimately uncertain. Alphatec is another company that has a broad product portfolio addressed to the United States market for fusion-based spinal disorder solutions [16]. Q1 financial results include total revenue of $30.1 million, up 27% year over year. Recent corporate highlights were an increased contribution from new products to 56% of Q2 2020 United States revenue, up from 22% in Q1 2019, and 48% in Q4 2019. However, on April 27, 2020 they terminated their prior agreement to acquire EOS imaging after considering the market effects of the COVID-19 pandemic. They also saw a significant decline in procedures in the last half of March 2020, as compared to earlier in Q1 2020, but remain hopeful for expected sales volumes to increase as early as Q2.

**Table 1:** Negative impact of COVID-19 on company sales in Q1 2020 compared to Q1 2019 sales

Companies	Q1 2019 sales (Million US Dollars)	Q1 2020 sales (Million US Dollars)	% difference Q1 2020/2019
Medtronic	691	480	-30.5%
Stryker	270	261	-3.3%
Globus	182.9	190.6	4.2%
NuVasive	274.78	259.88	-5. 4%
Johnson & Johnson	344	307	-10.8%
SeaSpine	36	36.1	0.3%
Orthofix	85.9	82.3	-4.2%
Zimmer	168	148	-11.9%
Alphatec	24.6	30.1	22.4%
Total	2077.18	1794.98	-13.6%

NuVasive, Inc. saw Q1 2020 total revenue of $259.9 million, a 5.4% decrease relative to $274.8 million in Q1 2019 [17]. On a constant currency basis, Q1 total revenue decreased by 5.1% compared to last year. As of March 31, 2020, the company had $512 million in cash and cash equivalents and had the ability to draw $550 million on their 2020 Facility. Additionally, they had outstanding $650 million aggregate principal amount of the 2021 Notes, which have a maturity date of March 2021, and $450 million aggregate principal amount of the 2025 Notes. The company’s ability to borrow under the 2020 facility is subject to remaining in compliance with their underlying financial agreements, which may prove difficult to comply with if the pandemic continues to negatively impact the health care system and business.

Globus Medical, Inc. shared worldwide Q1 sales of $190.6 million, an increase of 4.2% over Q1 2019, and 4.4% increase on a constant currency basis [18]. The company estimated the negative sales impact of COVID-19 to be $20 million in Q1. Q1 sales in the United States, including robotics, increased 7.4% as compared to Q1 of 2019, however international sales declined by 9.3% as compared to Q1 2019 on an as-reported basis. Despite the material impact of the current pandemic, Globus feels that they are in a strong position, including cash and borrowing capacity, to continue to sustain and grow the business after restrictions are lifted and elective surgeries resume.

Stryker Corporation reported the negative impact of the pandemic on consolidated net sales and detailed a lower-than-expected unit volume growth rate in all segments [14]. However, the company reported neurotechnology and spine net sales of $0.7 billion, a 0.7% increase in the quarter, and 1.5% in constant currency. Organic net sales rose 0.3% in Q1 from increased unit volume. Finally, SeaSpine's Q1 revenue was 36.1 million, comparable to Q1 2019, but the United States spinal implant revenue comprised 14.5%, reflecting a 3% drop from last year [19]. This decrease was attributed to lower procedure volumes and unit pricing, but the company was able to offset negative material impacts to some degree by a procedural mix shift to more thoracolumbar procedures, which generally gain more revenue per case relative to other procedures. The overall financial impact on spinal instrumentation companies will be largely based on future duration, spread, and intensity of the pandemic, which ultimately is uncertain and difficult to predict.

Impact on instrumentation company representatives

The reduction in revenue and the uncertainty of the duration and magnitude of the COVID-19 pandemic have directly impacted the work environment of spinal instrumentation companies’ employees. Like in many other industries, office-based employees are required to work remotely, and sales representatives can no longer visit the hospitals and clinics they used to frequent daily [20, 21]. The representatives now utilize videoconferencing to interact with customers and use remote learning modules to complete sales training [22-24]. Additionally, it has become more difficult to attend medical conferences not only due to the cancellation of many of those meetings, but also because employers have imposed restrictions on or completely canceled travel funding [21, 25-27].

Forced to reduce their operating expenses, many companies have instituted extreme measures that have inevitably affected the livelihood of their employees. Many workers face reduced working hours, tiered or flat salary cuts by up to 50% of base salary, marked compensation reductions, or being partially unemployed or laid off [20, 25, 28-32]. However, notwithstanding the economic challenges they are facing, some companies have implemented various strategies to mitigate the financial burden shouldered by their employees. For instance, emergency leave and sick time donation policies have been instated that allow workers who cannot report to their workplace and cannot work remotely to receive up to 30 days of pay. Moreover, financial assistance initiatives, such as voluntary salary-to-equity conversion programs, have been established to secure enough funds to aid employees impacted by the pandemic [33, 34].

Impact on surgeons

The COVID-19 pandemic has presented an unexpected situation for spinal surgeons. Surgeons normally tasked with managing the symptoms of spinal dysfunction and the decision to operate are now tasked with the additional burden of managing COVID-19. With the goal of reducing hospital admissions nationally, surgeons must heavily weigh the risks and benefits associated with immediate surgical intervention. The decision to operate may be clear and expected for acute emergencies, however, most spinal cases, especially those secondary to degenerative disease, lie in the gray area where the decision to operate urgently is not clear-cut. Opting for surgical intervention during the pandemic consumes the limited supply of health care resources and risks patient exposure. Many patients with spinal dysfunction have coexisting comorbidities making the patient population especially prone to the effects of COVID-19. For many patients, postponing surgery and managing symptoms for the foreseeable future is tolerable. However, the looming uncertainty of how long the COVID-19 pandemic may last further hinders the decision to provide surgical intervention. Furthermore, surgeons need to understand the risk of potential COVID-19 exposure to their self and the implication that it may impose on their future patients.

For surgeons, the inability to operate in a hospital setting can be a positive opportunity to broaden their scope of practice in the outpatient setting. Surgeons may use this unexpected time to begin providing services they normally refer to outside providers, such as epidural spinal injections. Additionally, with the drive toward less invasive procedures and technological advancements, many procedures such as kyphoplasty can be done in the office under the analgesic management of the surgeon. Many of these outpatient procedures offer relief to patients in a timely and effective manner while providing additional revenue to a surgeon’s practice. Additionally, as the pandemic limits the surgeon’s ability to offer surgical intervention to patients, the use of conservative treatment will be emphasized. Although this serves as a limitation in some facets, it is also an opportunity for surgeons to weigh the effectiveness of spinal instrumentation as the demand has increased in recent years. The National Hospital Discharge Survey demonstrated a 28-fold increase in anterior cervical decompression and spine fusion from 1990 to 2004 [35]. Additionally, from 2002 to 2007, there was a reported 15-fold increase in the treatment of lumbar spinal stenosis with complex fusions without a showing of increased disease complexity [36]. Prior studies suggest patients are offered high rates of unnecessary spinal surgeries, however, the extent to which this exists remains debated as symptoms may spontaneously improve.

Spinal surgeons are facing unprecedented times enduring the financial burden of the COVID-19 pandemic. The numbers are staggering, with one in five physicians having been furloughed or taken a pay cut [37]. Additionally, negative financial losses have been reported in 97% of medical practices and practice revenues overall are down 55% [38]. Hospitals and surgeons alike will face long-term consequences due to the pandemic. Surgical procedures are the strongest drivers of financial stability for both surgeons and hospitals. Fortunately, the abundance of postponed surgical cases in the pipeline provides a hopeful future to recover financially. It is important to develop a cohesive plan to optimize the return to surgical procedures and make up for lost revenue.

Future speculative analysis

Regaining normalcy in the post-COVID-19 phase will initially be driven by reestablishing surgical workflow. Emphasis must be placed on establishing a plan of action between surgeons and operating room leadership to ease the transition. With the backlog of surgeries, optimization and expanded operating room capacity are essential to progressing forward. This can be done by expanding surgical operating periods to include evenings and weekends or by increasing the number of active surgical suites. Additionally, less invasive procedures should be moved to dedicated rooms freeing surgical suites. Scheduling common surgical procedures together can increase efficiency by expediating room turnover and procedural preparation. Additionally, establishing surgical teams composed of staff with procedural knowledge allows for cases to run smoothly, ultimately allowing for more cases to proceed. Prehospital preparation for patients will be fundamental to ensuring surgical schedules run smoothly as new COVID-19 screenings will be an added requirement to surgical clearance. For spinal surgery, the added necessity of spinal instrumentation support poses an additional obstacle. Coordination and communication between surgeons, company representatives, and hospital leadership will be essential to establishing a smooth transition under the new constraints of COVID-19. The caseload will be strenuous on the system, however, understanding and collaboration between surgeons and staff will lead the road to recovery for both patients and practices.

The most notable impact COVID-19 may have on the spinal industry is the ultimate drive towards ambulatory surgical centers (ASC). With patients hesitant to self-expose to COVID-19 through hospital-based procedures, patients may feel more comfortable in ambulatory patient care centers. Many subspecialties have moved towards performing procedures in the ASC setting; however, spinal surgery remains largely hospital based. For many spinal surgeons, operating in ASC is out of their comfort zone, however, COVID-19 may be the spark needed to commit spinal surgery to the same evolution. Advancements in minimally invasive surgery and spinal instrumentation have allowed the transition out of the hospital to occur. Additionally, with the reduction in the required duration of post-surgical care and overnight staffing at ASCs, there is less need for hospital-based care. With the structure and resources geared towards maximizing surgical efficiency, it seems spinal surgery in ambulatory surgical centers has a bright future ahead. 

## Conclusions

The COVID-19 pandemic caused global implementation of strict measures to limit SARS-CoV-2 spread. These measures led to a drastic reduction in elective surgeries, which resulted in a ripple effect impacting different sectors of the spinal instrumentation industry. Hospitals and spinal surgeons have started following new treatment and communication guidelines. Although they have suffered from financial losses, the surgeons are given an opportunity to assess the effectiveness of different treatment modalities and explore new practice settings, for example, telemedicine. Spinal instrumentation companies have faced multiple severe economic challenges, which forced them to instate new policies to reduce their expenses and protect their employees, who are experiencing financial and workplace environment turmoil. Although the different sectors are currently facing dire financial hurdles, they are hopeful they will recover from these losses by following meticulous plans that will optimize the operational workflow as elective surgeries are permitted to resume.

## References

[1] (2020). Coronavirus disease 2019 (COVID- 19): situation report—37. https://www.who.int/docs/default-source/coronaviruse/situation-reports/20200226-sitrep-37-covid-19.pdf.

[2] Zhu N, Zhang D, Wang W (2020). A novel coronavirus from patients with pneumonia in China, 2019. N Engl J Med.

[3] (2020). Characteristics of operating room procedures in U.S. hospitals, 2011. https://www.hcup-us.ahrq.gov/reports/statbriefs/sb170-Operating-Room-Procedures-United-States-2011.jsp.

[4] COVIDSurg Collaborative (2020). Elective surgery cancellations due to the COVID-19 pandemic: global predictive modelling to inform surgical recovery plans. Br J Surg.

[5] Johnson & Johnson Reports 1st Quarter 2020 Results (2020). Johnson & Johnson 1st Quarter 2020 Results. http://johnsonandjohnson.gcs-web.com/static-files/ab8ded53-4e1e-400d-88d5-89ba1b5f3613.

[6] Louie PK, Harada GK, McCarthy MH (2020). The impact of COVID-19 pandemic on spine surgeons worldwide. Global Spine J.

[7] (2020). NASS guidance document on elective, emergent and urgent procedures. https://www.spine.org/Portals/0/assets/downloads/Publications/NASSInsider/NASSGuidanceDocument040320.pdf.

[8] (2020). When do elective surgeries start at US hospitals?. https://blog.definitivehc.com/when-do-elective-surgeries-start-at-us-hospitals.

[9] (2020). 30 states resuming elective surgeries. https://www.beckershospitalreview.com/cardiology/11-states-resuming-elective-surgeries.html.

[10] (2020). Local resumption of elective surgery guidance. https://www.facs.org/covid-19/clinical-guidance/resuming-elective-surgery.

[11] (2020). Roadmap from AHA, others for safely resuming elective surgery as COVID-19 curve flattens. https://www.aha.org/standardsguidelines/2020-04-17-roadmap-aha-others-safely-resuming-elective-surgery-covid-19-curve.

[12] Mummaneni PV, Theodosopoulos PV, Berger MS (2020). Letter: the coronavirus disease 2019 global pandemic: a neurosurgical treatment algorithm. Neurosurg.

[13] (2020). Updated II: How much the main spine companies have lost because of COVID-19 in Q1?. http://www.thespinemarketgroup.com/how-much-the-main-spine-companies-have-lost-because-of-covid-19-in-q1-2020/.

[14] (2020). Stryker Corporation, Form 10-Q. https://www.sec.gov/ix?doc=/Archives/edgar/data/310764/000031076420000069/syk10q3312020.htm.

[15] (2020). Zimmer Biomet Holdings, Inc., Form 10-Q. https://www.sec.gov/ix?doc=/Archives/edgar/data/1136869/000156459020024235/zbh-10q_20200331.htm.

[16] (2020). Alphatec Holdings, Inc., Form 10-Q. https://www.sec.gov/Archives/edgar/data/1350653/000156459020024330/atec-10q_20200331.htm.

[17] (2020). Nuvasive, Inc., Form 10-Q. https://www.sec.gov/ix?doc=/Archives/edgar/data/1142596/000156459020022043/nuva-10q_20200331.htm.

[18] (2020). Globus Medical, Inc., Form 10-Q. https://www.sec.gov/ix?doc=/Archives/edgar/data/1237831/000123783120000032/gmed-20200331x10q.htm.

[19] (2020). SeaSpine Holdings Corporation, Form 10-Q. https://www.sec.gov/Archives/edgar/data/1637761/000163776120000049/spne2020033110-q.htm.

[20] (2020). Medicrea is protected from the consequences of the COVID-19 pandemic [press release]. https://www.medicrea.com/wp-content/uploads/2020-03-19-Medicrea-Covid-19-EN-vdef.pdf.

[21] (2020). SeaSpine® provides preliminary results for first quarter 2020 and COVID-19 related business. https://www.seaspine.com/news/seaspine-provides-preliminary-results-for-first-quarter-2020-and-covid-19-related-business-update/.

[22] (2020). OrthoPediatrics Corp. provides COVID-19 business update. https://ir.orthopediatrics.com/news-releases/news-release-details/orthopediatrics-corp-provides-covid-19-business-update.

[23] (2020). COVID-19: implications for pharmaceutical and medical device companies. https://www.natlawreview.com/article/covid-19-implications-pharmaceutical-and-medical-device-companies.

[24] (2020). Re-entry guidance for health care facilities and medical device representatives. https://medtechresponds.com/wp-content/uploads/Re-entry-Guidance-for-Health-Care-Facilities-and-Medical-Device-Representatives.pdf.

[25] (2020). Exactech lays off 63, others face pay cuts. https://www.gainesville.com/business/20200323/exactech-lays-off-63-others-face-pay-cuts.

[26] (2020). COVID-19 update. https://www.stryker.com/content/dam/stryker/corporate/policies/en-us/COVID19_CustomerFAQs_Final.

[27] (2020). More medical conferences fall to coronavirus. https://www.medscape.com/viewarticle/926359.

[28] (2020). Stryker Corporation, current report pursuant to section 13 or 15(d) of the securities exchange act of 1934. https://www.sec.gov/ix?doc=/Archives/edgar/data/310764/000031076420000061/syk8-k4272020.htm.

[29] (2020). Zimmer Biomet provides update on COVID-19 impact. https://investor.zimmerbiomet.com/news-and-events/news/2020/04-06-2020-210914865.

[30] (2020). Medacta announces pay cuts to BoD and GEM members and publishes 2020 AGM invitation. https://www.medacta.com/EN/press.

[31] (2020). Orthofix announces preliminary first quarter 2020 results and provides business update. http://ir.orthofix.com/news-releases/news-release-details/orthofix-announces-preliminary-first-quarter-2020-results-and.

[32] (2020). Boston Scientific cutting hours and pay as revenue drops. https://www.bostonglobe.com/2020/04/02/business/boston-scientific-cuts-pay-36000-workers-citing-revenue-drop-because-coronavirus/..

[33] (2020). Medtronic provides update on COVID-19 pandemic response and impact. http://investorrelations.medtronic.com/news-releases/news-release-details/medtronic-provides-update-covid-19-pandemic-response-and-impact.

[34] (2020). ATEC announces preliminary first quarter 2020 revenue results. https://atecspine.com/atec-announces-preliminary-first-quarter-2020-revenue-results/.

[35] Marawar S, Girardi FP, Sama AA (2010). National trends in anterior cervical fusion procedures. Spine.

[36] Watts C (2014). Neurosurgery: a profession or a technical trade?. Surg Neurol Int.

[37] (2020). Physicians and COVID-19. https://www.merritthawkins.com/uploadedFiles/Corona_Physician_Survey_Merritt_Hawkins_Report.pdf.

[38] (2020). COVID-19 financial impact on medical practices. https://stateofreform.com/wp-content/uploads/2020/04/MGMA.pdf.

